# Computational Construction of a Single-Chain Bi-Paratopic Antibody Allosterically Inhibiting TCR-Staphylococcal Enterotoxin B Binding

**DOI:** 10.3389/fimmu.2021.732938

**Published:** 2021-11-23

**Authors:** Ganggang Bai, Yanhong Ge, Yuhong Su, Shuo Chen, Xingcheng Zeng, Huixia Lu, Buyong Ma

**Affiliations:** ^1^ Engineering Research Center of Cell & Therapeutic Antibody (MOE), School of Pharmacy, Shanghai Jiao Tong University, Shanghai, China; ^2^ Molcell Biodesign, Inc., Frederick, MD, United States

**Keywords:** bispecific antibody, staphylococcal enterotoxin B, superantigen, TCR, antibody design, molecular dynamics simulation, allostery

## Abstract

Staphylococcal enterotoxin B (SEB) simultaneously crosslinks MHC class II antigen and TCR, promoting proliferation of T cells and releasing a large number of toxic cytokines. In this report, we computationally examined the possibility of using a single-chain biparatopic bispecific antibody to target SEB and prevent TCR binding. The design was inspired by the observation that mixing two anti-SEB antibodies 14G8 and 6D3 can block SEB-TCR activation, and we used 14G8-6D3-SEB tertiary crystal structure as a template. Twelve simulation systems were constructed to systematically examine the effects of the designed bispecific scFV MB102a, including isolated SEB, MB102a with different linkers, MB102a-SEB complex, MB102a-SEB-TCRβ complex, MB102a-SEB-TCR-MHC II complex, and MB102a-SEB-MHC II. Our all atom molecular dynamics simulations (total 18,900 ns) confirmed that the designed single-chain bispecific antibody may allosterically prevent SEB-TCRβ chain binding and inhibit SEB-TCR-MHC II formation. Subsequent analysis indicated that the binding of scFV to SEB correlates with SEB-TCR binding site motion and weakens SEB-TCR interactions.

## Introduction

Bispecific antibodies contain two different antigen-binding sites in one molecule. The concept of combining two antigen-recognizing elements into a single molecule to simultaneously bind to two distinct targets was first used in 1960 (1), and it has gained much attention recently in the development of novel therapies to treat cancer, autoimmunity, neurodegeneration, and infections (2–4). One of the most popular approaches is the bispecific T-Cell engaging antibodies for cancer therapy, so called BiTE for ‘‘bispecific T-cell engager’’ (5). There are about 20 different architectures to construct the bispecific antibodies (2, 4, 6), and the connecting two scFV (single-chain variable fragment) represents a successful and promising immunotherapy platform (7). The bispecific scFV can be commonly used to target two separate targets to activate T-cell in cancer immune-therapy (8), or bind two protomers in HIV-1 envelope glycoproteins trimer complex (9). Biparatopic bispecific antibodies recognize two different epitopes on one molecule and are promising formats for the development of next-generation antibody therapeutics (10–13). Therefore, it is interesting to examine a novel approach to use a biparatopic antibody to target Staphylococcal enterotoxin B (SEB), a small single domain protein with at least four non-overlapping epitopes.

Staphylococcus aureus belongs to gram-positive bacterium and has become a major threat to health (14). Staphylococcal enterotoxin B (SEB) is one of the best characterized and is a superantigens because of its ability of simultaneously binding to MHC class II antigen and TCR to form a complex, promoting the proliferation of T cells and releasing a large number of cytokines (15). With a poisoning dose of merely 0.4 ng/kg, SEB has been listed in the biological weapons list (16). Many SEB antibodies have been found to play a protective role in the SEB-induced diseases (17–21). Among them, mAb 20B1, mAb 14G8 and mAb 6D3 have three non-overlap SEB epitope regions (22). 20B1 binds on the TCR binding site, preventing the formation of MHC-TCR-SEB complex, thus it has the more prominent neutralization (21, 23). 6D3 and 14G8 alone can only achieve lower protection and even no protection respectively, even with higher dose treatment (14), since their epitopes are far away from TCR binding site. However, combinations of any two of 20B1, 6D3 and 14G8 enhance the protective effect. The combined action of 6D3 and 14G8 may induce SEB to produce subtle conformational changes, which may prevent SEB-TCR interaction and enhance SEB neutralization (22).

In this research article, we computationally investigated the effects of a designed single-chain biparatopic antibody derived from antibodies 6D3 and 14G8. Extensive molecular dynamics simulations have shown that the binding of the designed bispecific scFv with SEB allosterically prevents SEB-TCR association and formation of SEB-MHC-TCR complexes. Subsequent analysis indicated that the binding of scFV to SEB correlates with SEB-TCR binding site motion and weakens SEB-TCR interactions.

## Materials and Methods

### Construction of Biparatopic Bispecific scFV and Simulation System Preparation

**Figure 1:**
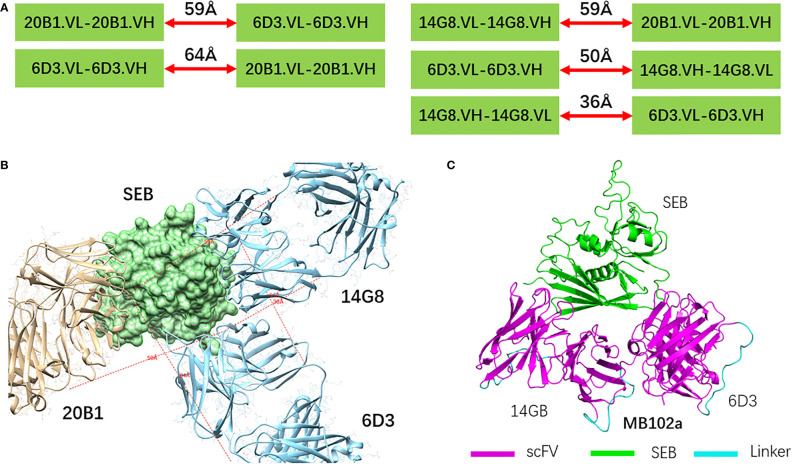
Construction of bispecific scFVs **(A)** Five possible combinations considered to construct bispecific scFVs from three antibodies 20B1, 6D3 and 14G8. The minimal linker length are indicated using red arrows. **(B)** By superimposing of SEB-20B1 (PDB 4RGM) and SEB-6D3-14G8 (PDB 4RGN) structures on SEB to probe possible linker connecting nearby scFVs. SEB molecule is represented as green surface **(C)** Designed bispecific scFV antibody MB102a by connecting variable domains of 14G8FV and 6D3FV.

Five possible combinations were considered to construct bispecific scFVs from three antibodies 20B1, 6D3 and 14G8 (**Figure 1**). By superimposing SEB-20B1 (PDB 4RGM) and SEB-6D3-14G8 (PDB 4RGN) structures on SEB, we examined the distances needed to connect two scFVs. As can be seen in **Figure 1**, 6D3 and 14G8 are close to each and 20B1 has longer distances to either 6D3 or 14G8. Connecting of 20B1 with 6D3 or 14G8 requires linkers at least longer than 60Å. For the architectures to connect 6D3 and 14G8, we found that the 14G8FV and 6D3FV can be connected using a linker as shorter as 3X: (SGGGG)3 in the connecting order of (14G8.VH-3X-14G8.VL)-3X-(6D3.VL-3X-6D3.VH). The resulting bispecific scFV will be called as MB102a scFV.

Six simulation systems were constructed to systematically examine the effects of the MB102a scFV on SEB-TCR crosslinking (**Table 1**): isolated SEB, isolated MB102a scFV-3x, MB102a scFV-3x-SEB complex, MB102a scFV-3x-SEB-TCRβ complex, MB102a scFV-3x-SEB-TCR-MHC II complex, and MB102a scFV-3x-SEB-MHC II. The structure of isolated SEB APO was directly obtained from the crystal structure of SEB (PDB 1SE4) (24). The scFV·SEB structures were obtained by first removing constant regions of 14G8_Fab_ and 6D3_Fab_ from SEB-6D3-14G8 ternary complex crystal structure (PDB 4RGN) (22) and then linking the 14G8_FV_ and 6D3_FV_ as MB102a scFV with different linkers. The isolated scFV APO structure was obtained by manually removing the antigen from the bound structure. The structure of scFV-3x-SEB-TCRβ complex was merged from scFV-SEB with SEB-TCRβ complex (PDB:1SBB). The tetrameric (scFV-3x·SEB·TCR·MHC II) complex was constructed by merging binary (scFV-3x·SEB) complex with ternary complex SEB·TCR·MHC II structure (PDB ID: 4C56). The ternary (scFV-3x·SEB·MHC II) complexes was obtained by removing TCR from tetrameric scFV·SEB·TCR·MHC II complex.

**Table 1:** Details about the simulated antibody-antigen complexes.

System	Template PDB	Simulation Time	Total atoms	Water
SEB(apo)*	4RGN	1000ns	65280	20383
scFV-3x (apo)*	4RGN	1000ns	109180	33904
scFV-3x-SEB*	4RGN	1000ns	153520	47321
scFV-3x-SEB-TCR*	4RGN and 1SBB	220ns	237931	74189
**SEB-TCR**	1SBB	1000ns	134967	42396
**scFV-3x-SEB-TCR-MHC** II *	4RGN and 4C56	470ns	359544	111486
**scFV-3x-SEB-MHC** II *	4RGN and 4C56	1000ns	230577	70889
**SEB-TCR-MHC** II	4C56	1000ns	248472	76982
scFV-4x-SEB*	4RGN	1000ns	156457	48331
scFV-5x-SEB*	4RGN	1000ns	154997	47832
scFV-5x-LB(apo)*	4RGN	1000ns	122220	38156
scFV-5x-LB-SEB*	4RGN	1000ns	156129	48112

*These simulations were performed twice.

In order to examine the effects of different linkers on the bispecific scFV-SEB binding, we also simulated three additional constructions with 4X: (SGGGG)4, 5X: (SGGGG)5, and 5X-LB. In the 4X and 5X system, the linkers connecting VH and VL of 6D3 and14G8 are still 3X, but that connecting 6D3 and14G8 changed to 4X and 5X, respectively. In the 5X-LB system, the linkers connecting VH and VL of 6D3 and14G8 changed to 15 amino acid fragment GSTSGSGKSSEGKGG (25), and that connecting 6D3 and 14G8 is 25 amino acid 205C linker LSADDAKKDAAKKDDAKKDDAKKDL (26).

### MD Simulation Protocols

The conserved disulfide bonds of systems were constructed according to PDB files. For the light chain, the heavy chain, and the antigen, the N termini and C termini were charged as $\text{N}{\text{H}}_{3}^{+}$ and COO^−^ groups, respectively. The missing residues are reconstructed using the CHARMM-GUI input generator (27). The systems were then solvated by TIP3 water molecules with minimal margin of 15 Å from any protein atom to any edge of water box. Sodium and chloride ions were added to neutralize the system to a total concentration of ~150 mM by vmd software (28). The resulting solvated systems were energy-minimized for 50000 steepest descent steps, followed by an additional 50000 conjugate gradient steps, where all atoms could move. In the heating stage, each system was gradually heated to 50K and then to 250K. In the production stage, all simulations were performed using the NPT ensemble at 300 K, with timestep of 2fs. The particle mesh Ewald (PME) method was used to calculate the electrostatic interaction, and the van der Waals interactions were calculated using a cutoff of 8 Å. All MD simulations were performed using the amber20 software (29) and last 1000ns. MD trajectories were saved by every 0.1ns for analysis. A summary of all simulation systems is given in **Table 1**. All simulated systems (except the stable references complexes SEB-TCR and SEB-TCR-MHC II) were simulated twice using different starting conditions.

### MD Simulation Analysis

**RMSD, RMSF calculation:** The root mean squared deviation (RMSD) and root mean square fluctuation (RMSF) for the backbone of each structure are calculated by VMD. We use the chothia numbering scheme (30) to label complementarity-determining regions (CDRs).

**Contact map analysis:** We construct the protein (14G8, SEB, and 6D3) contact map by software ConAn (31) to analysis the residue-residue interaction.

**Correlation analysis**: Correlations between all the residues were analyzed for the entire 1000-ns MD trajectory (10000 frames) using the normalized covariance of the motion of protein residues (32), ranging from -1 to 1. If two residues move in the same (opposite) direction in most the frames, the motion is considered as (anti-)correlated, and the correlation value is close to 1 or -1. If the correlation value between two residues is close to zero, they are generally uncorrelated. The correlations evaluation were performed using program CARMA (33).

## Results

### MB102a scFV Binds SEB in the Way Identical to SEB-6D3-14G8 Complex Crystal Structure

In order to test the convergence of simulation, we first performed 1000 ns of isolated SEB and then compare the RMSF of SEB from simulation with the experimental B-factors of two SEB crystal structures (PDB: 1SE4 and 3SEB). The experimental B-factors were converted to RMSF using the following relationship: $B=\frac{8\pi^{2}RMSF^{2}}{3}$ . As can be seen in **Supplementary Figure 1A**, RMSF from MD simulations essentially reproduced the residue fluctuations corresponding to the RMSF converted from experimental B-factors, indicating excellent simulation convergence. For the scFV part, we repeated the scFV-3x-apo simulation with a different minimization and heating steps and added a scFV-5x-LB-apo simulation. As can be seen in **Supplementary Figure 1B**, three simulations have similar RMSD, even though they have different RMSD trajectories. However, the RMSF values for the heavy and light chains of 14G8 and 6D3 in the three simulations indicated certain variations from different simulations (**Supplementary Figures 1C-F**). It is known that antibody CDR loop re-arrangements occur in the micro-to-millisecond timescale, and it needs more extensive simulations to fully capture the CDR conformation landscapes (34). Nevertheless, except a few jumps of RMSF in some CDR loops, the overall features from 3 independent simulation of scFV-3x-apo and scFV-5x-LB-apo agree well.

**Figure 2:**
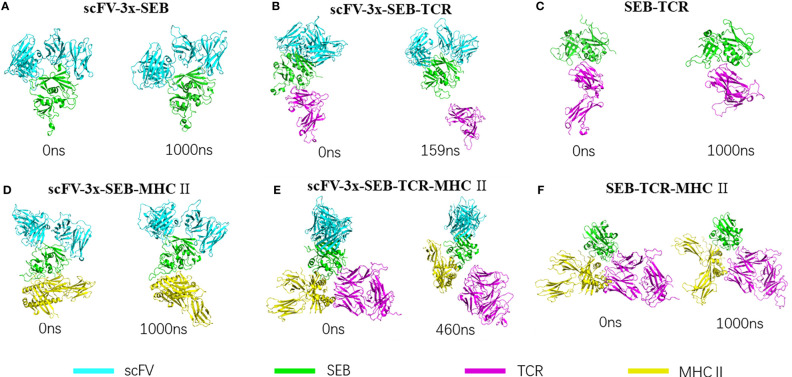
| Bispecific scFV antibody MB102a (scFV-3x) promote disassociation of SEB-TCR, illustrated by starting and ending conformations of four complex simulated. **(A)** Stable scFV-3x-SEB complex. **(B)** Binding of MB102a (scFV-3x) break up SEB-TCRβ chain interaction. Simulation stopped at 159 ns after TCRβ chain disassociated. **(C)** SEB-TCRβ complex is stable throughout 1000 ns simulation. **(D)** scFV-3x-SEB-MHC II complex is stable throughout 1000 ns simulation. **(E)** Binding of MB102a leads TCR molecule to dissociate from SEB- MHC II. Simulation stopped at 460 ns after TCRβ chain disassociated. **(F)** SEB-MHC II complex is stable throughout 1000 ns simulation.

**Figure 3:**
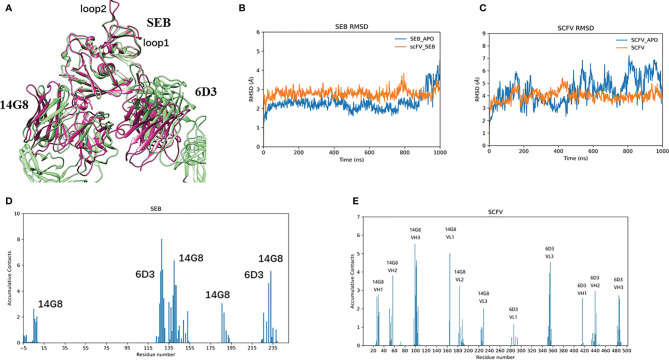
MB102a scFV from stable complex with SEB. **(A)** Superimposing of SEB from final snapshot (1000 ns) of the scFV-SEB with that in the PDB 4RGB structure. **(B, C)** are RMSD trajectories of SEB and MB102a scFV, respectively. The contact frequencies of SEB **(D)** with MB102a scFV **(E)** are plotted.

**Figure 2** lists snapshots of the conformations of six complexes at their starting and the end of simulations: scFV-3x-SEB, scFV-3x-SEB-TCR, SEB-TCR, scFV-3x-SEB-MHC II, scFV-3x-SEB-TCR-MHC II, and SEB-TCR-MHC II. Throughout 1000 ns simulation time, two control system SEB-TCR and SEB-TCR-MHC II remain stable. The designed scFV stays bound with SEB in the way identical to its parent SEB-6D3-14G8 complex (PDB 4RGN, **Figure 2A**). Since there is no crystal structure for the scFV system, we use FAB system as a reference. Superimposing of SEB from final snapshot (1000 ns) of the scFV-SEB with that in the PDB 4RGN structure indicated that RMSD of all 214 comparable atom pairs is as small as 1.91Å, and scFV is only slightly twisted from their positions in the crystal structure (**Figure 3A**). Using the starting conformation used in MD simulation as a reference, we can see that the RMSD trajectory of SEB portion stay around 3 Å (**Figure 3B**). Interestingly, the RMSD trajectory of SEB in the bound form is higher than that in isolated state. The conformations of scFV in bound form have much smaller RMSD comparing that in free state (**Figure 3C**), indicating certain dynamic coupling among scFV and SEB antigen.

**Figure 4:**
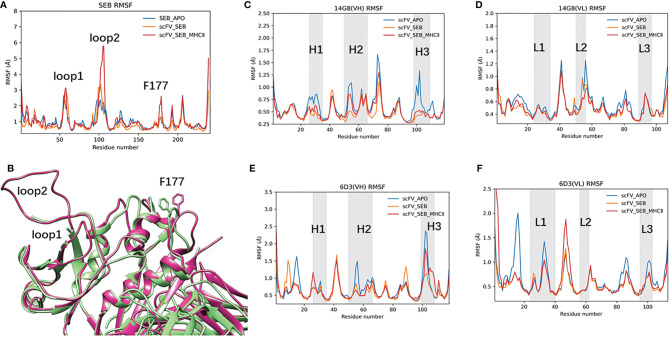
Binding of Bispecific scFV antibody on SEB leads to changes of RMSF for all interaction partners, illustrated by the change of RMSFs of different protein chains in three states: free state, MB102a-SEB complex, and MB102a-SEB-MHC II complex. **(A)** RMSF of SEB. **(B)** superimposing of the crystal structure of SEB (green ribbon) with the final snapshot in MB102a-SEB complex (purple ribbon). **(C)** 14G8 VH chain. **(D)** 14G8 VL chain. **(E)** 6D3 VH chain. **(F)** 6D3 VL chain.

The RMSF plots for the SEB-scFV complexes are shown in **Figure 4**. In crystal structures, residues 97 to 107 and 109 are poorly defined even at 1.5 Å (3SEB) to 1.9 Å (1SE4) resolution and have been modelled as alanine residues (loop2, **Figures 4A, B**). Consistently, these loop97-107 residues have high B-factors. A nearby loop of residue 54-61 also has high B-factors in the crystal structures. In our simulation of isolated SEB molecule, we also observed high residue flexibilities for these two loops. (**Figures 4A, B**). Consistent with solvent-accessible surface area (SASA) trajectories, the 14G8 scFV has smaller RMSF in bound state than in free state (**Figures 4C, D**). However, there are several regions in the 6D3 that has higher RMSF in the bound state (**Figures 4D, F**).

**Figure 5:**
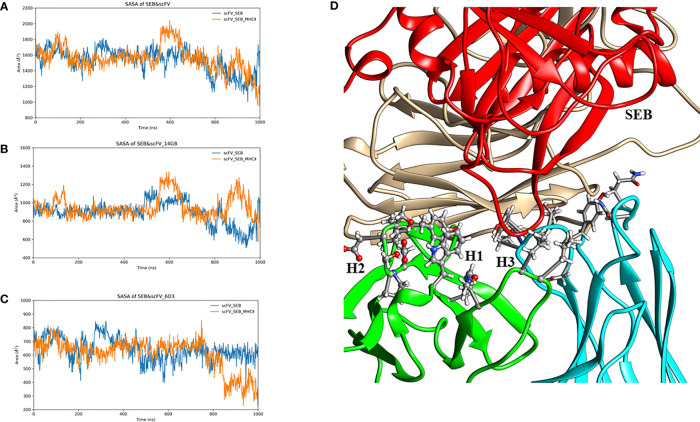
14G8 interact with SEB stronger than 6D3. Contact surface areas of SEB with all MB102a scFV **(A)**, with 14G8 variable domain **(B)**, and with 6D3 variable domain **(C)**. **(D)** illustrated of SEB interaction with 6D3 variable domain in the last snapshot of 1000 ns simulation.

In the crystal structure (22), the total SASA buried between SEB and 14G8Fab is around 928 -941 Å^2^, and that the total solvent-accessible surface area of 6D3Fab covered by SEB is 833 Å^2^. In our simulation of scFV-SEB complex, we found that the total SASA between the SEB and scFV fluctuates between 1400-1800 Å^2^ in first 500 ns, and then has higher dynamics in the second phase of simulation from 500 – 1000 ns (**Figure 5A**). **Supplementary Figure 2** lists the distance trajectories for several key interaction between SEB and 14G8. The interactions of SEB^R135^-14G8^D31^, SEB^R135^-14G8^Y32^, SEB^D139^-14G8^Y58^, SEB^k141^-14G8^F94^, and SEB^E231^-14G8^Y50^ are stable throughout 1000 ns simulation; while SEB^k188^-14G8^Y100^ and SEB^Y232^-14G8^Y100^ distances start fluctuating from 700 to 1000 ns, leading to the contact area surface fluctuation.

The interaction of 6D3 with SEB is weaker than the 14G8-SEB interaction (22), and the SASA trajectory of 6D3 with SEB fall mostly around 600 Å^2^ (**Figures 5B, C**). The residue contact frequencies between antibodies and SEB antigen are shown in **Figures 3D, E**. These contact patches are also the same as that in the crystal structure. The decrease of contact area between SEB and 6D3 mostly come from the H1 region (**Figure 5D**), which interact with Lys153/Lys226 of SEB in the crystal structure. From distance trajectories we can also see that residues D127, K128, and Y129 of SEB mostly maintain interactions with their partners, while R130 and R153 fluctuate away from their interaction patterns in crystal structure (**Supplementary Figure 3**).

### Binding of Bispecific scFV With SEB Allosteric Prevent SEB- TCRβ Chain Interaction

The structure of the complex between a mouse TCR β chain and SEB at 2.4 Å resolution revealed that Vβ CDR2 and FR3 account for the majority of contacts with the SEB (PDB 1SBB) (35). The crystal structure contains two asymmetrical copies of SEB-TCR β chain complex, indicating intrinsic flexibility of SEB-TCR β chain recognition (35). We simulated a hypothetic bispecific scFV-SEB-TCRβ chain complex to investigate the effect of bispecific scFV binding on the SEB-TCR β chain interaction and found that the TCRβ chain quickly dissociated from SEB in the hypothetic scFV-SEB-TCRβ complex (**Figure 2B**). The simulation stopped at 220ns after the TCRβ chain completely separates from scFV-SEB. We monitored the distance trajectories for several important interactions between SEB-TCRβ chain to investigate the allosteric effects of the antibody-SEB binding (**Supplementary Figure 4**). TCRβ chain forms important interactions with SEB^F177^, however with variations already in two sets of molecules in the SEB-TCRβ crystal structure. For example, SEB has four van der Waals contacts with TCRβ^H47^ in one complex, but only two in another copy of complex. As can be seen in **Figures 4A, B**, bispecific scFV binding allosterically causes the SEB^F177^ flip away from crystal structure position. In the simulation of the hypothetic scFV-SEB-TCRβ complex, the SEB^F177^ immediately increase its distances from TCRβ^H47^, TCRβ^Y65^, and TCRβ^K66^. At the 60 ns, these distance experience another large increases, leading to the perturbation of SEB^Y90^- TCRβ^A52^ interaction, which start to separate around 120 ns (**Supplementary Figure 4**). The SEB^Y91^- TCRβ^Y50^ also break up at around 120 ns. The whole TCRβ chain eventually lost all interaction with SEB at around 160 ns. With a different starting simulation condition, we again observed the leaving of TCRβ chain from SEB at 530 ns (**Table 2**).

**Table 2:** The interaction energy and dissociated time of SEB-TCR.

System	Interaction Energy (kcal/mol)	dissociated time (ns)
scFV-3x-SEB-TCR_1	SEB-TCR 1.72 ± 1.98	160
scFV-3x-SEB-TCR_2	SEB-TCR 1.25 ± 1.01	530
SEB-TCR	SEB-TCR 0.40 ± 1.88	–
scFV-3x-SEB-TCR-MHC II_1	(SEB-MHC)-TCR -0.62 ± 3.07	460
scFV-3x-SEB-TCR-MHC II_2	(SEB-MHC)-TCR -1.19 ± 2.76	>1000
SEB-TCR-MHC II	(SEB-MHC)-TCR -6.17 ± 2.96	–

The interaction between 14.3.d TCR VβCβ with wild-type SEB has a Kd of 140 μM. We used Foldx program to calculate the interaction energy between SEB and TCRβ chain using conformers obtained in the MD simulations. The binding energy is 0.40 ± 1.88 kcal/mol. While one would expect a negative value, the small positive repulsive binding energy and large standard deviation reflected weak SEB-TCRβ interaction. As can be seen in **Table 2**, the binding of scFV to SEB push the SEB- TCRβ to be more repulsive in both simulation replicates.

### Binding of Bispecific scFV Weakens TCR Interactions in SEB-TCR-MHC II Complex

The structure of a bacterial SEB, bound to a human class II histocompatibility complex molecule (HLA-DR1) has shown that no large conformational changes occur upon complex formation in either the DR1 or the enterotoxin B molecules (36). Surprisingly, in the ternary complex of SEB in complex with TCR and MHC class II, the SEB-TCRβ chain and SEB- MHC II portions are almost identical to their individual complexes and still allow TCRα chain to contact MHC and enable SEB to initiate a peptide-independent activation of T cells (37). We simulated two hypothetic bispecific scFV bound complexes (scFV-3x-SEB-MHC II and scFV-3x-SEB-TCR-MHC II) to investigate the possible inhibitory effects on T cell activation.

scFV-SEB-MHC II system stays as one complex through 1000 ns simulation, indicating that designed bispecific scFV does not prevent SEB-MHC II binding (**Figure 2D**). Comparing with the starting conformation, the hemagglutinin peptide is the most stable chain, with RMSD around 2Å, SEB and MHC α chain have RMSD around 2-4 Å, scFV and MHC β chain have RMSD higher than 4 Å (**Supplementary Figure 5A**). In the scFV-SEB-MHC II complex, the loop2 and F177 loop in SEB have larger residue fluctuation than in either isolated SEB or scFV-SEB (**Figure 4A**). 14G8 variable domain has slightly higher RMSF in scFV-SEB-MHC II than in the scFV-SEB complex, but still lower than those in free MB102a scFV (**Figures 4B, C**). The contact surface area between the 14G8 variable domain and SEB in the scFV-SEB-MHC II complex sometime increases to 1200 Å^2^, much higher than 928 -941 Å^2^ in crystal structure. 6D3 variable domain experienced large allosteric perturbation, and the segments connecting L1 and L2 has sharper RMSF increase around residue 50 (**Figure 4F**). The contact surface area between the 6D3 variable domain and SEB in the scFV-SEB-MHC II complex has a large drop around 800 ns and stabilized to have around 300 Å^2^ contact area at the end of simulation (**Figure 5C**).

It is interesting to know if additional interaction of TCRα chain with MHC in the hypothetic scFV-SEB-TCR-MHC II can stabilize the SEB-TCR interaction. Simulation of scFV-SEB-TCR-MHC II system shows that TCR also breaks from scFV-SEB-MHC II, but at a slower pace than in the case of scFV-SEB-TCRβ chain system. As shown in **Figure 2E**, TCR completely dissociate at 460 ns. Throughout the simulation of scFV-SEB-TCR-MHC II system, the contact areas between 14G8-SEB (**Supplementary Figure 5B**) and 6D3-SEB (**Supplementary Figure 5C**) are stable. As discussed in the last section, the large fluctuation of the F177 loop triggers TCRβ separating from scFV-SEB complex. The situation is the same for the scFV-SEB-TCR-MHC II system, where SEB^F177^ quickly increase its distances with TCRβ^Y49^ and TCRβ^V68^ (**Supplementary Figure 6**, upper panel). SEB-TCRβ contact starts to break at around 350 ns. While TCRα-MHC contact constantly fluctuate when MD simulation starts, it only quickly disassociates after SEB-TCRβ has no contact (**Supplementary Figure 6**, lower panel). However, in the second simulation run the TCR does not break out from scFV-SEB-TCR-MHC II system within 1000 ns simulation time. Still, we see that scFV weaken TCR-(SEB-MHC) interaction in both simulation runs. Using 10000 conformations obtained from MD simulations, the average Foldx interaction energy between TCR and SEB-MHC is -6.17 ± 2.96 kcal/mol in SEB-TCR-MHC II ternary complex. However, the interaction decreases to -0.62 ± 3.07 and -1.19 ± 2.76 kcal/mol for the first and second run, respectively.

### Connecting Linkers Have Subtle Effects on scFV-SEB Interactions

**Figure 6:**
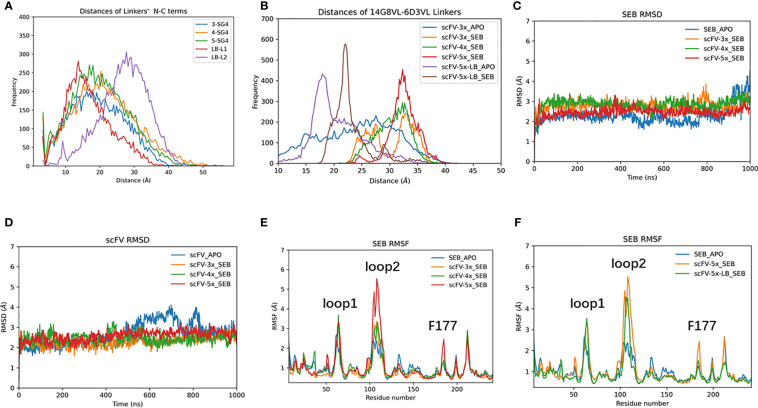
The effects of connecting loop of scFV. **(A)** end-end distance distribution of isolated loop peptide in solution during 1000 ns MD simulations. **(B)** Comparable distance distribution of connecting loop within scFVs during 1000 ns MD simulations. **(C)** RMSD trajectories of SEB. **(D)** RMSD trajectories of scFV, which is average of RMSD of 14G8 and 6D3 VH/VL chains at each frame. **(E, F)** RMSF plots of SEB domain in different complexes.

We simulated scFV constructs with different linkers. To examine the flexibilities of linkers, we have run 1000 ns MD simulations for each of the linker peptides corresponding to (SG4)3 (3X), (SG4)4 (4X), (SG4)5 (5X), GSTSGSGKSSEGKGG (LB1), and 205C linker (LB2, 5X-LB) sequences. As can be seen in **Figure 6A**, the peptide end-end distances distribution of 3X, 4X, and 5X are very similar, indicating that SG4 repeat linkers are extremely flexible and random. The popular 205C linker (LB2) tends to have a longer end-end distance than (SG4)5 (5X). However, after fused into scFV as connecting linkers, their corresponding distance distributions are totally different from those as free peptides (**Figure 6B**). Consequently, these linkers may have subtle effects on scFV’s dynamic and binding properties.

The scFV-3x, scFV-4x, and scFV-5x in their SEB complexes have similar RMSD trajectories (**Figures 6C, D**). Analysis of residue contacts between scFV constructs and SEB also shows conserved patterns (**Supplementary Figures 7**, **8**). As described in earlier sections, 14G8 has more contact residues and larger contact area with SEB than 6D3 has (**Supplementary Figures 8, 9**). Among the four different scFV-SEB complexes (scFV-3x, scFV-4x, scFV-5x, and scFV-5x-LB), scFV-4x-SEB and scFV-5x-LB-SEB are more stable than scFV-3x-SEB and scFV-5x-SEB.

The RMSF values of SEB and scFVs in different complexes also revealed slight differences due to different linkers used. As can be seen in **Figures 6E, F**, the RMSF values of loop2 are much higher for scFV-5x-SEB and scFV-5x-LB-SEB than scFV-3x-SEB and scFV-4x-SEB. The RMSF plots of scFV-3x, scFV-4x, and scFV-5x are in **Supplementary Figure 10**, and scFV-5x-LB’s RMSF plots are in **Supplementary Figure 11**. Overall, scFV-5x-SEB has higher RMSF values than scFV-3x-SEB and scFV-4x-SEB systems. In certain regions, the RMSF values of scFV-5x-SEB are higher than isolated scFV-3x (scFV-3x), not showing rigidification due to antigen binding. scFV-5x-LB binding has clear rigidification effects, and RMSF values of scFV-5x-LB-SEB are generally smaller than those of scFV-5x-LB-apo (**Supplementary Figure 11**).

### Allosteric Residue Correlations of Bispecific Antibody – SEB Complexes

**Figure 7:**
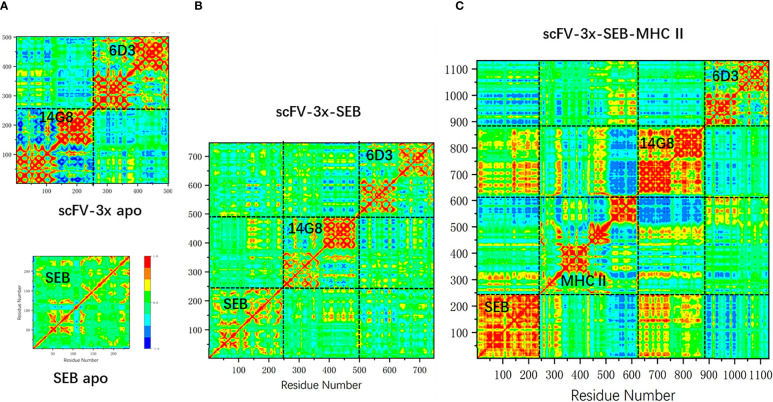
The change of covariance matrix of SEB and scFV-3x in different binding states. Red (blue) color corresponds to positive (negative) correlation in which residues move in the same (opposite) direction. **(A)** Isolated MB102a (scFV-3x, upper panel) and SEB (lower panel). **(B)** scFV-3x-SEB complex. **(C)** scFV-3x-SEB-MHC II complex.

We have observed that the RMSF of SEB loop2 is very sensitive to antibody and MHC II binding (**Figures 4A, B**, **6E, F**). In **Figure 7**, we systematically examined the changes of covariance matrix for SEB, scFV-3x, and scFV-3x-SEB-MHC II. In the isolated apo state, the long-range residue correlations in SEB are weak (**Figure 7A**). For scFV-3x, the residue motion correlations within each domain are strong, probably due to immunoglobulin fold. The VH and VL chains in 14G8 have negative motion correlation (since the off-diagonal block are mostly blue), and the corresponding correlations in 6D3 are slightly positive. The binding of scFV-3x and SEB increase motion correlation within SEB considerably, and 14G8 VH and VL chain changed from negative correlation to moderate positive (**Figure 7B**). Apparently, antigen binding synchronized motions of VH and VL chains. With binding of three proteins, SEB in scFV-3x-SEB-MHC II is rigidified in most regions except a few loops. As a result, the motion correlation within the SEB changed to strongly positive, and 14G8 in the scFV-3x-SEB-MHC II experienced similar effects (**Figure 7C**).

**Figure 8:**
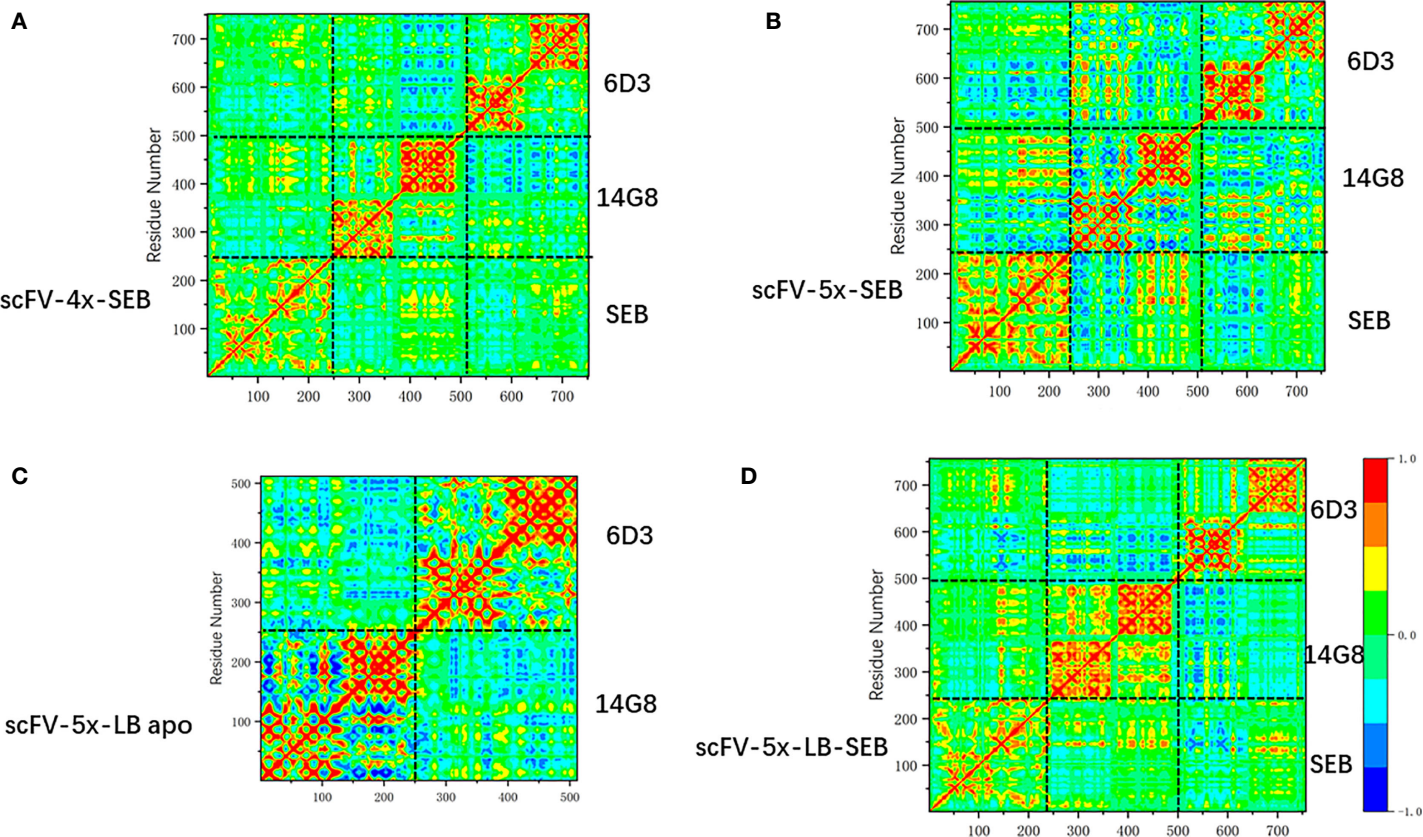
The change of covariance matrix scFVs with different linkers. Red (blue) color corresponds to positive (negative) correlation in which residues move in the same (opposite) direction. **(A)** scFV-4x-SEB complex. **(B)** scFV-5x-SEB complex. **(C)** scFV-5x-LB in isolated state. **(D)** scFV-5x-LB-SEB complex.

Change of linkers in bispecific scFV constructs has moderate effects on amino acid correlations in the scFV-SEB complexes. In **Figure 8** we compare the covariance matrixes of scFV-4x-SEB, scFV-5x-SEB, and scFV-5x-LB-SEB. One may notice that the covariance matrixes of scFV-3x-SEB (**Figure 7B**), scFV-4x-SEB, and scFV-5x-LB-SEB are very similar; while scFV-5x-SEB system has stronger overall motion correlations.

As discussed earlier, Phe177 is one of the most important residues for SEB-TCR binding. In order to reveal the allosteric communications of Phe177 with other amino acid regions in SEB and bound scFV, we compare the long range covariances of Phe177 in isolated SEB, scFV-3x-SEB, and scFV-5x-LB-SEB. **Table 3** lists residues with moderate correlation with Phe177, but separated by at least 18 Å. Essentially there is no long rang correlation of Phe177 in the isolated SEB. However, Phe177 allosterically correlates with many regions in SEB and bound scFV domains. In the SEB part, the highest correlations locate around 14G8 binding epitopes. 6D3 binding epitope residue are also allosterically correlated. Consistently, the CDR loops of 14G8 and 6D3 also show moderated correlation with Phe177. These correlations are similar in scFV-3x-SEB and scFV-5x-LB-SEB. While there are slight variations, these correlations from the second run of scFV-5x-LB-SEB essentially reproduced the results from first run. Clearly, these allosteric correlations could underly the mechanism of prevent SEB-TCR interaction through the binding of scFVs on the SEB.

**Figure 9:**
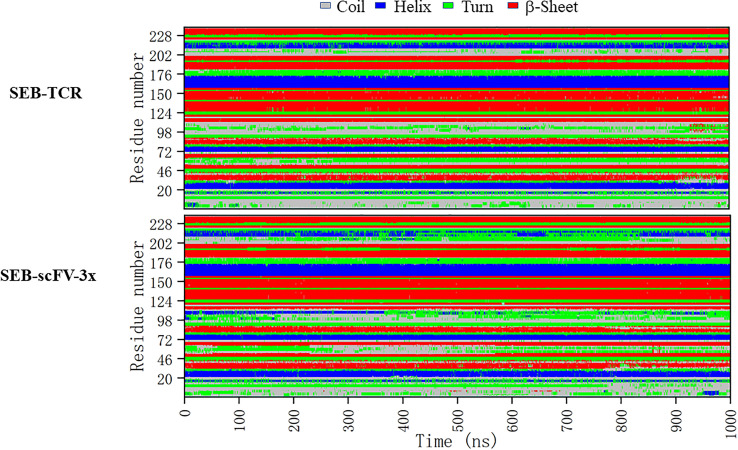
Comparison of secondary structure changes of SEB-TCR and SEB-scFV-3x during 1000 ns simulation. Regions around loop2 residue 95-105 and residues 202-228 has the largest differences.

In order to see if these correlated regions have differences in secondary structure dynamics, we compare the secondary structure trajectories of SEB-TCR and SEB-scFV-3x (**Figure 9**). Regions around loop2 residue 95-105 and residues 202-228 has the largest differences. Interestingly, two residues (213-214), which have large correlation with Phe177 in **Table 3**, are in the 202-228 region. In the SEB-TCR complex, there is more helical content for the residues 202-228. However, in the SEB-scFV-3x complex, the residues 202-228 has more turn characteristics (**Figure 9**), implying that secondary structure change could relate to the allosteric residue correlations.

**Table 3:** Allosteric correlations of Phe177 with SEB and scFV amino acid residues.

SEB-apo			scFV-3x-SEB			scFV-5x-LB-SEB			
Residues	Correlation	Distance(Å)	Residues	Correlation	Distance(Å)	Residues	Correlation(Run1)	Correlation(Run2)	Distance(Å)
10-11	-0.01/0.04	18-20	10-11	0.3/0.38	18-20	11-11	0.23/0.34	0.29/0.35	18-20
31-32	-0.10/-0.17	19-22	31-32	0.22/0.32	19-22	31-32	0.30/0.34	0.20/0.25	19-22
72-91	-0.12/0.05	21-43	72-91	-0.37/0.33	21-43	72-91	-0.29/0.35	-0.21/0.34	21-43
119-123^a^	-0.02/0.06	35-43	119-123 ^a^	-0.23/-0.34	35-43	119-123 ^a^	-0.17/-0.23	-0.26/-0.33	35-43
135-146^b^	-0.02/-0.11	19-24	135-146 ^b^	0.24/0.50	19-24	135-146 ^b^	0.31/0.51	0.13/0.35	19-24
161-164	-0.01/0.06	19-21	161-164	0.28/0.40	19-21	161-164	0.39/0.44	0.21/0.27	19-21
213-214	-0.01/-0.02	18-21	213-214	0.31/0.35	18-21	213-214	0.34/0.38	0.37/0.39	18-21
232-238^b^	-0.02/0.22	13-21	232-238 ^b^	0.35/0.66	13-21	232-238 ^b^	0.42/0.58	0.22/0.43	13-21
			14G8VH2	0.12/0.34	21-28	14G8VH2	0.29/0.41	-0.03/0.29	21-28
			14G8VL2	-0.26/-0.34	43-54	14G8VL2	-0.06/-0.16	-0.08/-0.14	43-54
			14G8VL3	0.05/0.21	30-32	14G8VL3	0.25/0.31	-0.22/0.10	30-32
			6D3VL1	-0.3/-0.35	33-47	6D3VL1	-0.11/-0.32	-0.39/0.18	33-47

^a^6D3 binding epitope; ^b^14G8 binding epitope.

## Discussion

Use of antibody cocktails has received more and more attention in pharmaceutical development, such as Inmazeb — a mixture of three monoclonal antibodies again Ebola virus (38). The approaches using antibody cocktail may avoid the virus escape by RNA virus or other drug resistance that is inherent in monotherapy approaches (39). The bispecific antibodies (2, 4, 6) represent a different approach. Besides their unique biological effects, bispecific antibodies are more time and resource efficient, without need to make two different clinical-grade antibodies.

Bi-paratopic bispecific antibodies can target two nearby epitopes as in the case of HIV-1 envelope glycoproteins (9, 40) and Bi-paratopic and multivalent VH domains targeting SARS-CoV-2 (11). The small toxin antigen studied here, staphylococcal enterotoxin B (SEB), may represent the closest epitopes one may expect for a bispecific antibody to bind. The superantigen SEB have at least four non-overlapping protein binding sites. However, our simulations indicate that not all of the four binding sites can be occupied simultaneously due to allosteric effects upon protein-protein interaction. As a result, the binding of our designed single-chain bispecific antibody would allosterically prevent SEB-TCR association, thus block TCR activation.

Allostery is an intrinsic property of all dynamic proteins (41, 42), and it is important to examine allosteric effects for biological drugs. Due to the large size, the allosteric effects of biological drug could be more effective than small molecule allosteric drugs. Several allosteric antibodies have been published. For examples, antibody mAb7 inhibits the glucagon GCGR receptor through a unique allosteric mechanism (43); and an allosteric anti-tryptase antibody can treat mast cell-mediated severe asthma (44). Besides the allosteric effects on the antigen, different regions in antibody also have allosteric communications (45–47), including scFv (48). A strategy to identify linker-based modules for the allosteric regulation of antibody-antigen binding affinities of different scFVs was also proposed (49). Our current study also found that linkers have subtle effects on the dynamics and binding properties of our constructed scFVs.

Computational approaches have been proved to be important methods in antibody design (50), to understand antibody dynamics in antigen binding and affinity maturation, especially using extensive simulations and Markov-state model (51–53). Our current approaches, using multiple MD microsecond simulations to study different comparable systems also captured essential features of antibody-antigen interactions near experimental determined antibody-antigen template structures. However, due to highly dynamic nature of antibody structure, the time scale of the studied conformation change could be much longer than used in our simulations. The force fields and other conformation search limitations in current MD simulation protocol used will affect simulation results. Previous studies indicated that for the free antibody in solution, the CDR conformation and dynamics (54) need more extensive simulations (51–53). Nevertheless, our current results provided an effective approach to test antibody design and allosteric mechanisms accompanying antibody-antigen interactions.

Superantigen SEB represents a protein with important immunology significances and biophysical interests. As a single domain protein with only 250 amino acids, the SEB has at least four non-overlapping binding sites to interact with TCR, MHC, and antibodies. While the allosteric communication network with SEB domain is latent in the isolated state, it responds to various protein bindings. In future, it is interesting to delineate further underlying biophysical mechanisms and to investigate other similar superantigen systems.

## Data Availability Statement

The original contributions presented in the study are included in the article/**Supplementary Material**. Further inquiries can be directed to the corresponding author.

## Author Contributions

GB and BM designed the experiments and wrote the manuscript. GB, YG, YS, SC, XZ, and HL conducted experiments and analyzed the data. All authors contributed to the article and approved the submitted version.

## Conflict of Interest

Author BM was employed by company Molcell Biodesign, Inc.

The remaining authors declare that the research was conducted in the absence of any commercial or financial relationships that could be construed as a potential conflict of interest.

## Publisher’s Note

All claims expressed in this article are solely those of the authors and do not necessarily represent those of their affiliated organizations, or those of the publisher, the editors and the reviewers. Any product that may be evaluated in this article, or claim that may be made by its manufacturer, is not guaranteed or endorsed by the publisher.
