# Supplementary material for: Computational Construction of a Single-Chain Bi-Paratopic Antibody Allosterically Inhibiting TCR-Staphylococcal Enterotoxin B Binding
Source: Front Immunol. 2021 Nov 23;12:732938. doi: 10.3389/fimmu.2021.732938 (PMC8649926; doi:10.3389/fimmu.2021.732938)
Supplement: Supplementary file 1 [file DataSheet_1.pdf]

# *Supplementary Material*

# Computational Construction of a Single-chain Bi-paratopic Antibody Allosterically Inhibiting TCR-Staphylococcal Enterotoxin B Binding

## 1.1 Supplementary Figures

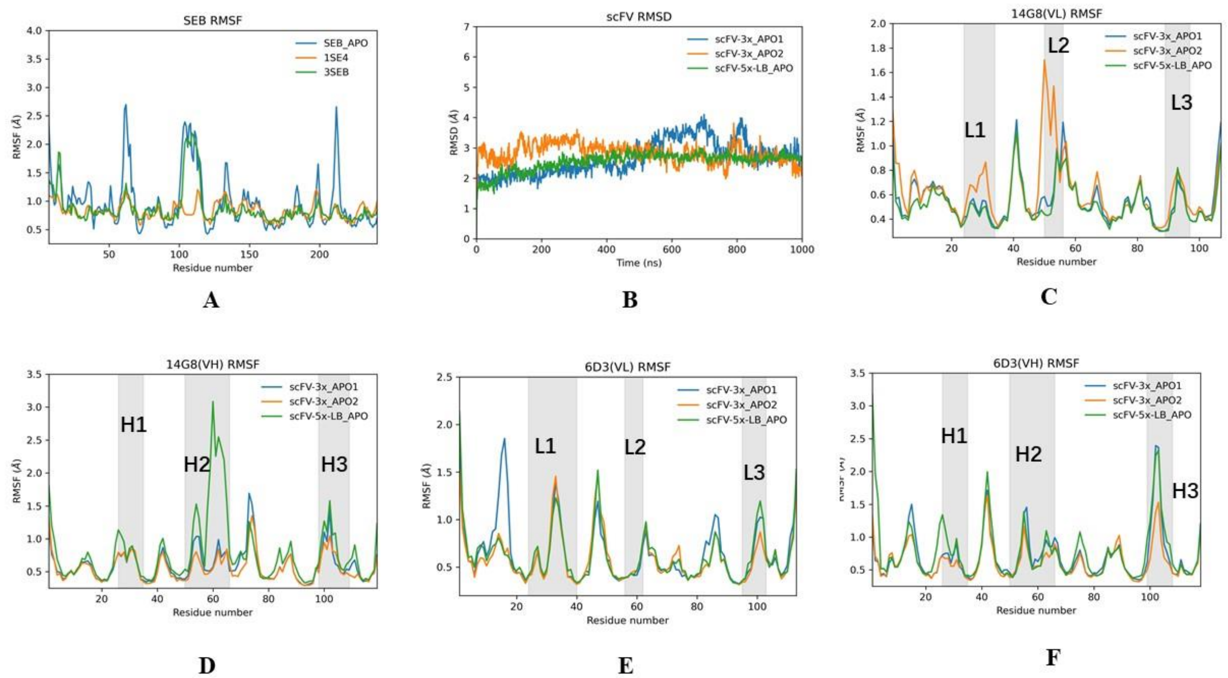


**Supplementary Figure 1.** RMSD and RMSF from repeated simulations. (A) comparison of the RMSF of SEB from simulation with the experimental RMSF converted by B-factor of two SEB crystal structures (1SE4, 3SEB). (B) RMSD trajectories of scFV, which is the average of RMSD of 14G8 and 6D3 VH/VL chains at each frame. (C-F) RMSF of VH and VL chains of two 14G8 and 6D3.

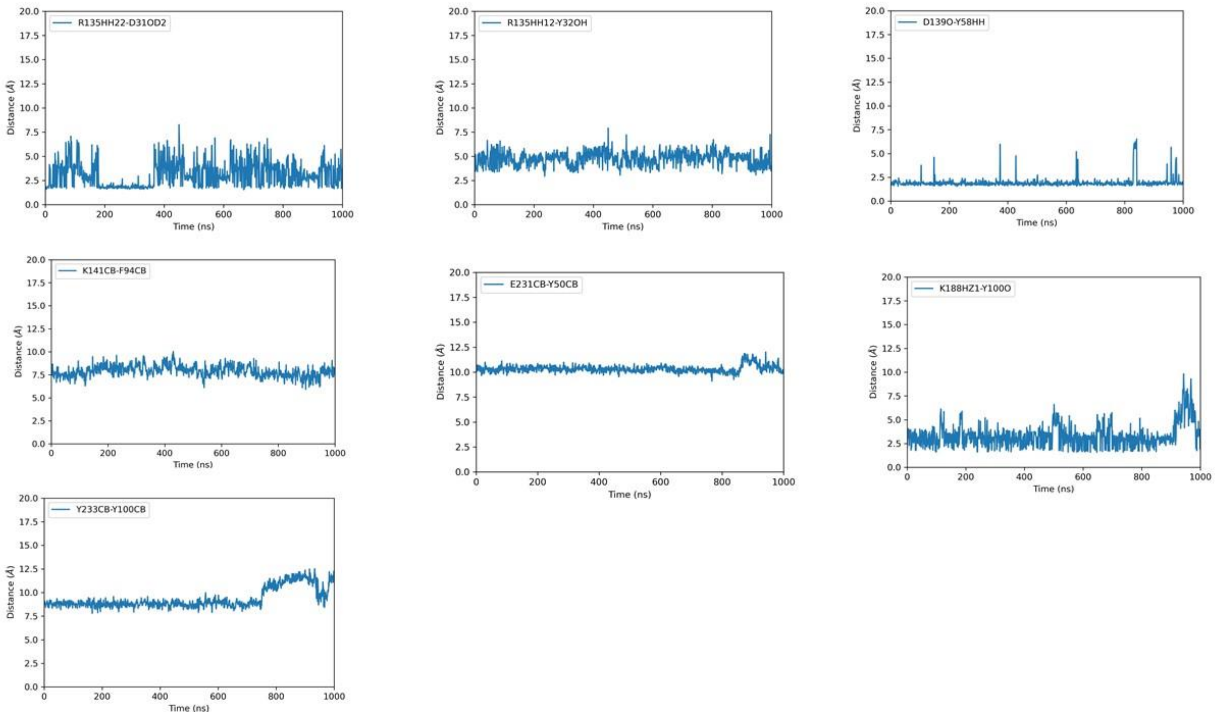


**Supplementary Figure 2.** Atomic distance trajectory for selected contact residues between SEB and 14G8 variable domain MB102a scFV-SEB complex.

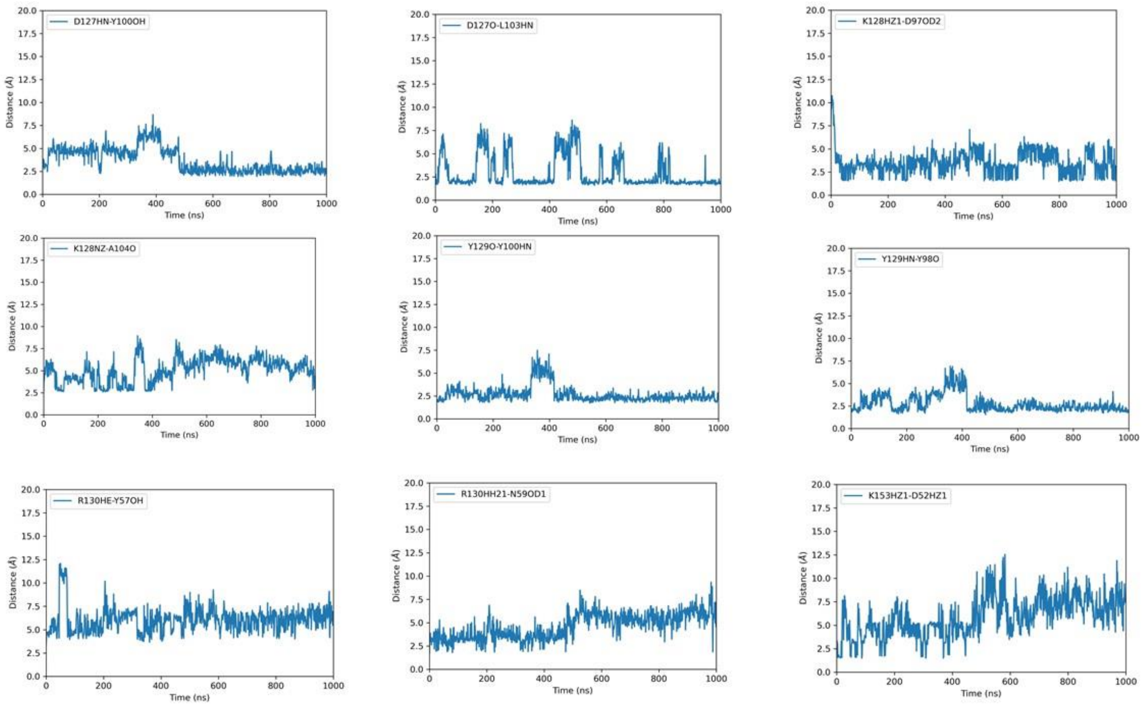


**Supplementary Figure 3.** Atomic distance trajectory for selected contact residues between SEB and 6D3 variable domain in MB102a scFV-SEB complex.

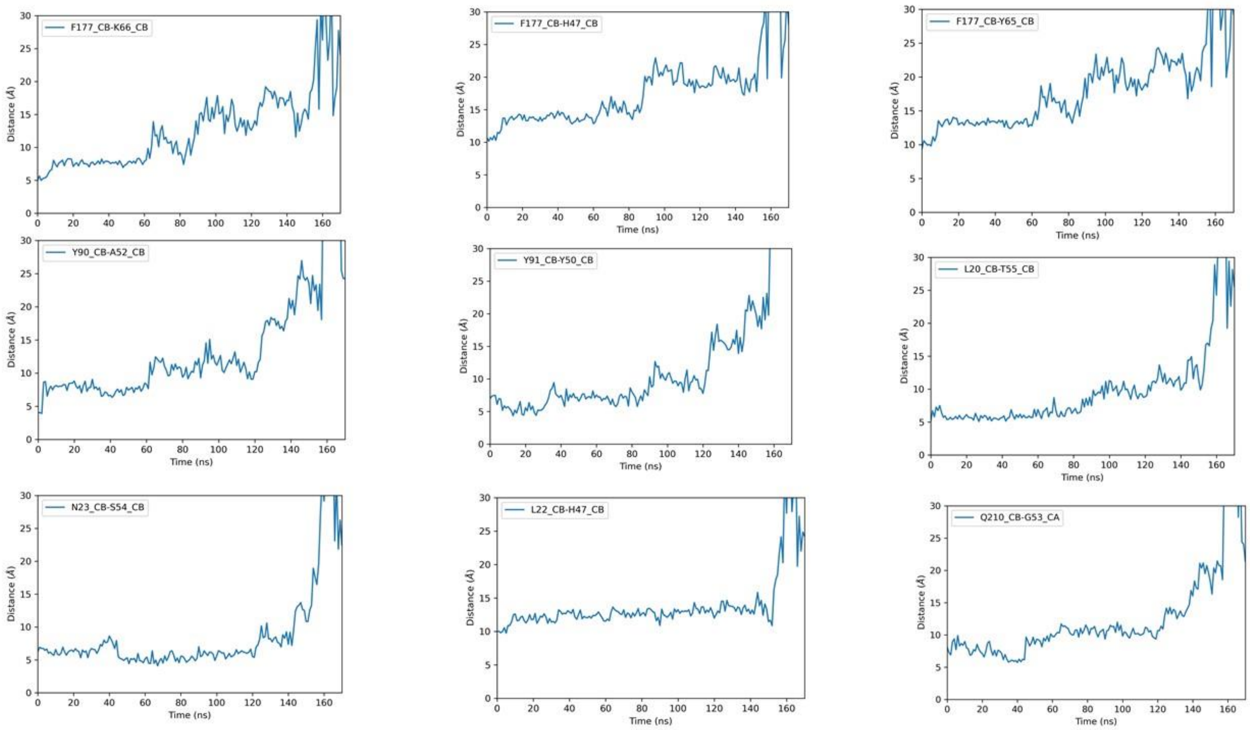


**Supplementary Figure 4.** Atomic distance trajectory for selected contact residues between SEB and TCRβ chain in the first simulation of MB102a scFV-SEB-TCRβ chain complex.

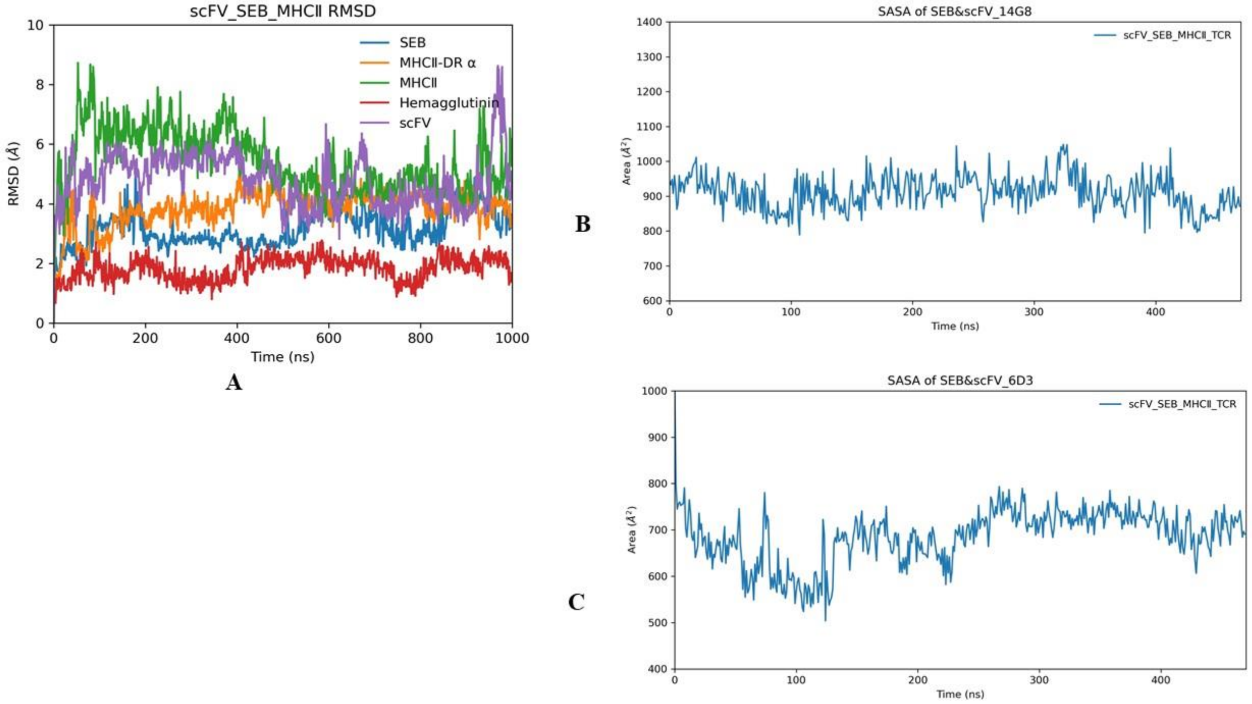


**Supplementary Figure 5.** (A) RMSD trajectories of different chains in MB102a scFV-SEB-MHC II complex. While TCR dissociate from scFV-SEB-TCR-MHC II complex, both 14G8 variable domain (B), and with 6D3 variable domain (C) have stable contact with SEB.

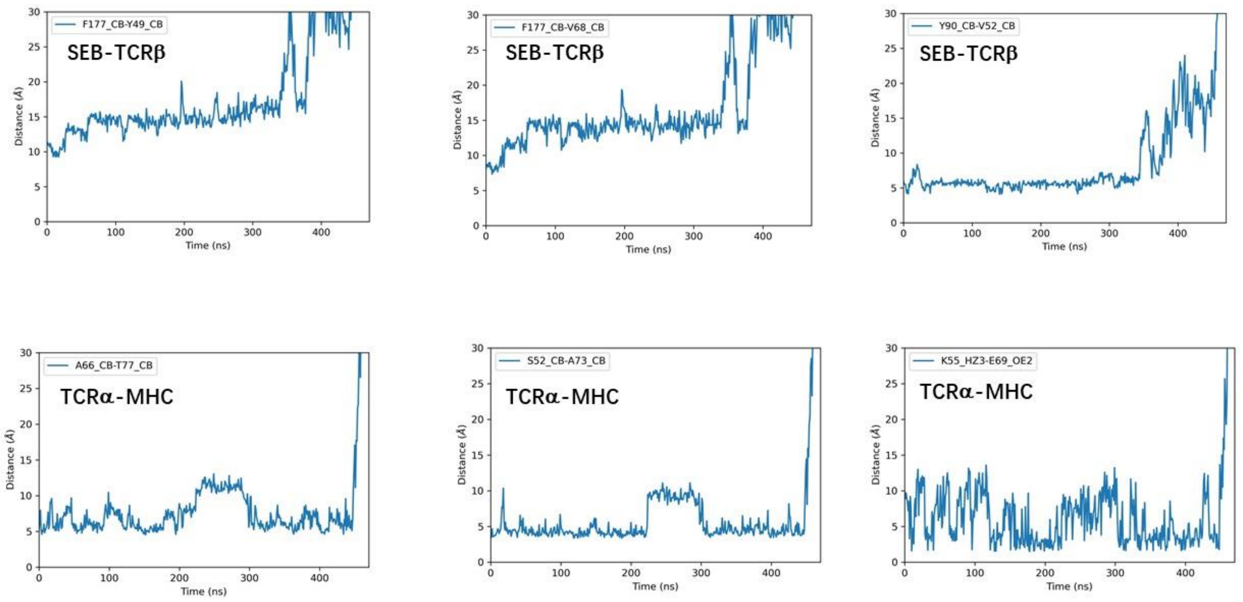


**Supplementary Figure 6.** Atomic distance trajectory for selected contact residues between SEB and TCRβ chain (Upper panel) and between MHC and TCRα chain (lower panel) in the simulation of MB102a scFV-SEB-TCR–MHC II complex.

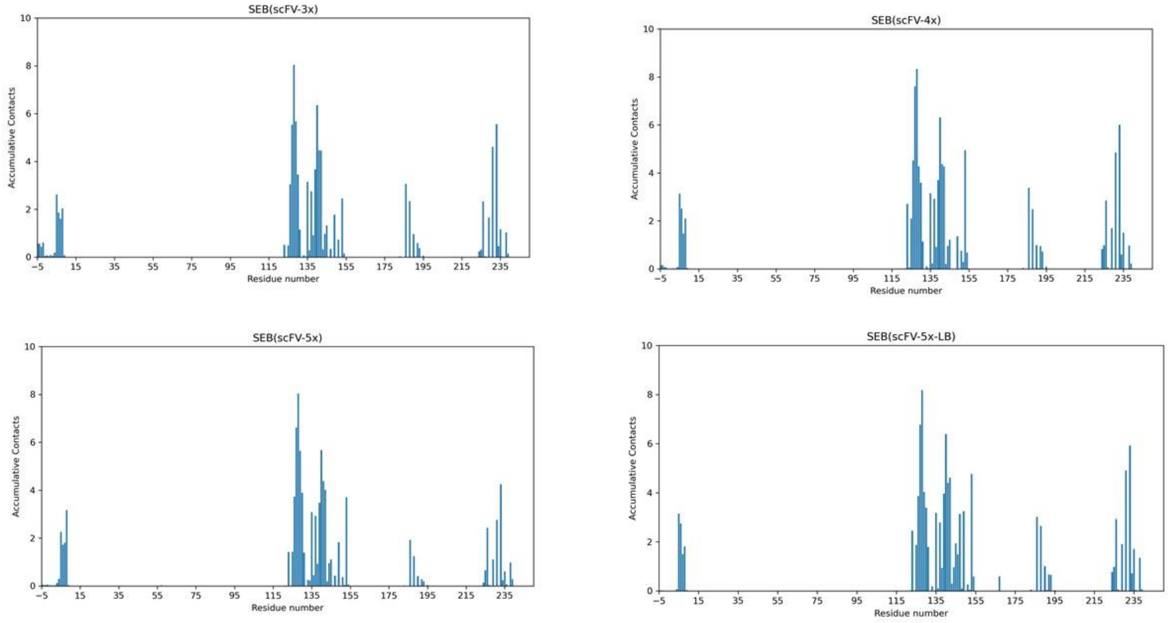


**Supplementary Figure 7.** SEB has similar residue contact pattern with scFVs of different linkers.

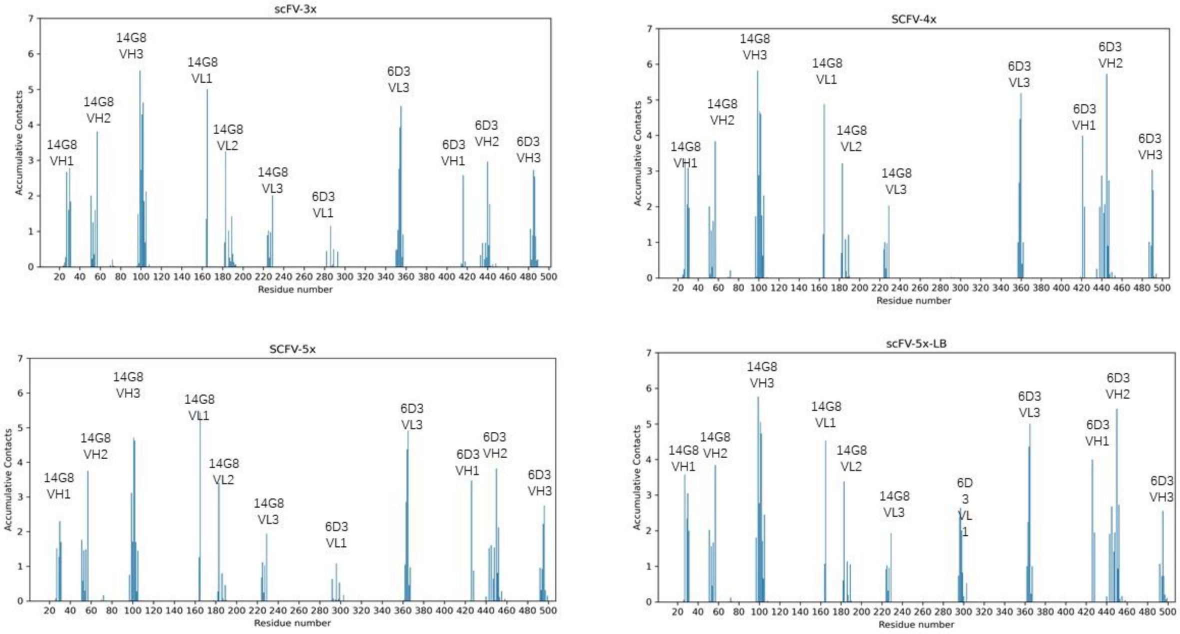


**Supplementary Figure 8.** scFVs of different linkers have similar SEB contact pattern.

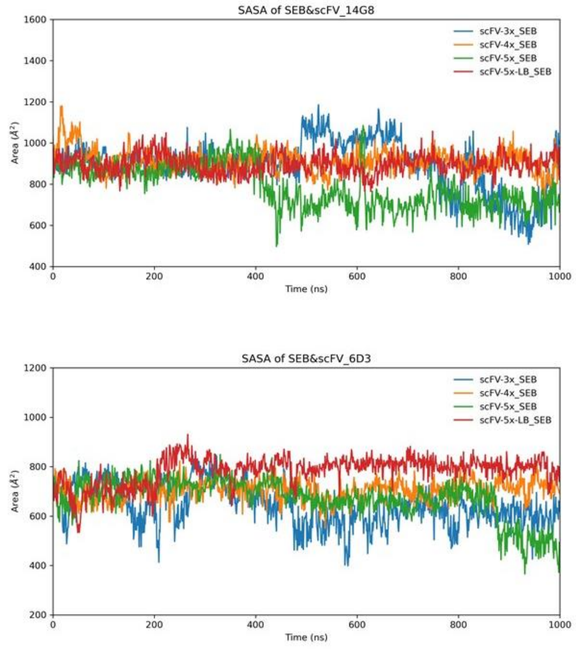


**Supplementary Figure 9. scFV-4x-SEB and scFV-5x-LB-SEB have more stable SEB contact area than scFV-3x-SEB and scFV-5x-SEB.**

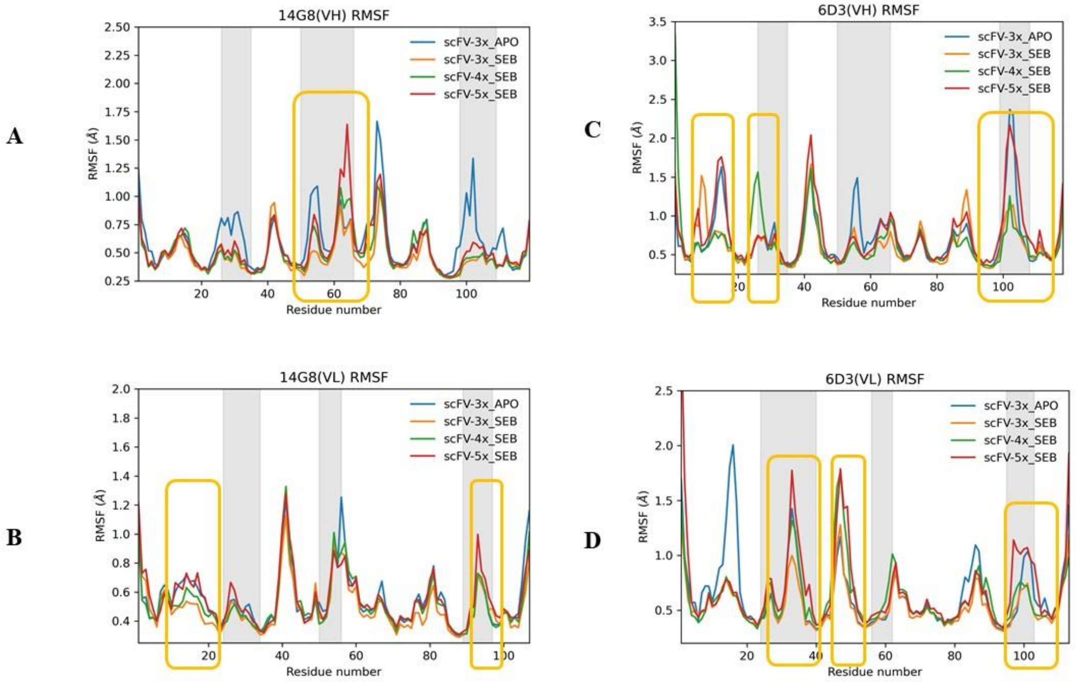


**Supplementary Figure 10. Comparison of RMSF plot of scFV with different linker length.**

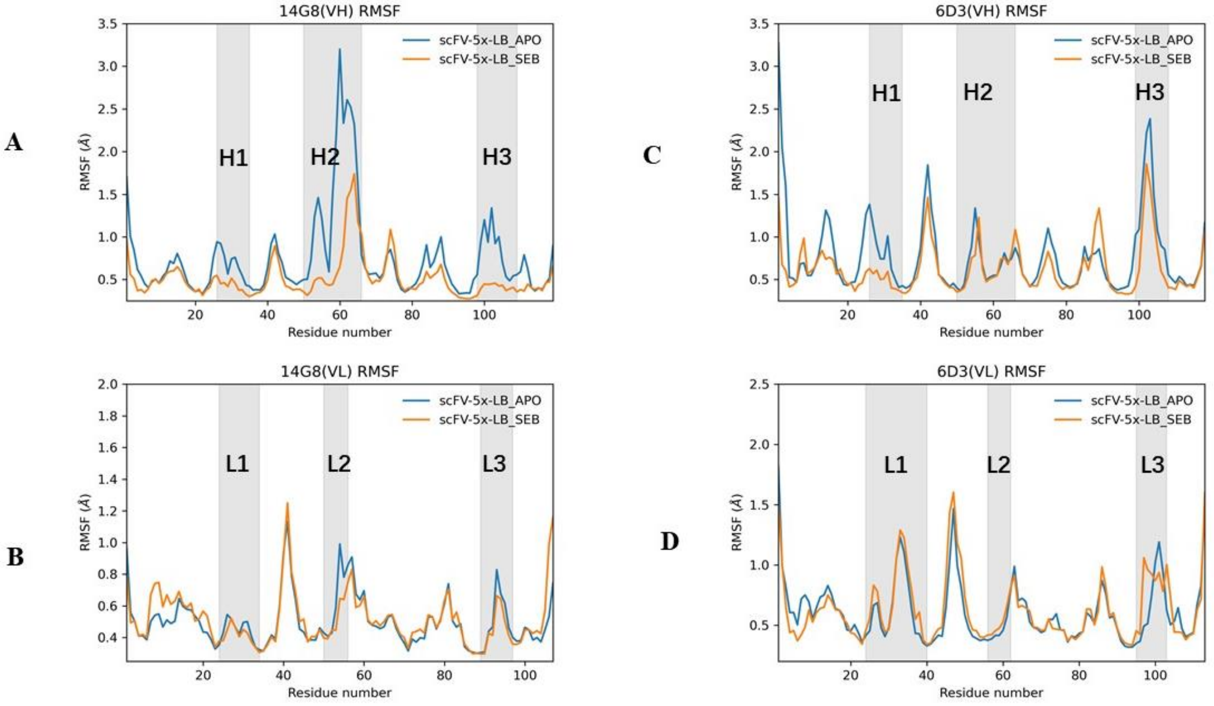


**Supplementary Figure 11. Binding with SEB rigidify CDRs of scFV-5x-LB.**

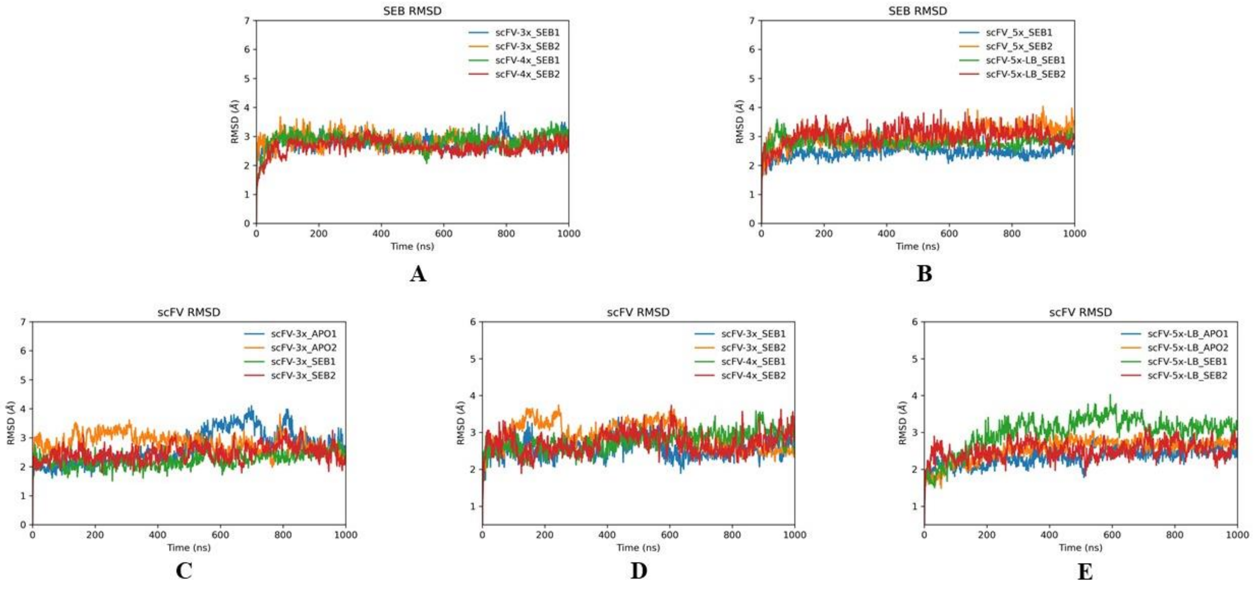


**Supplementary Figure 12. Comparison of the RMSD from repeated simulations.**

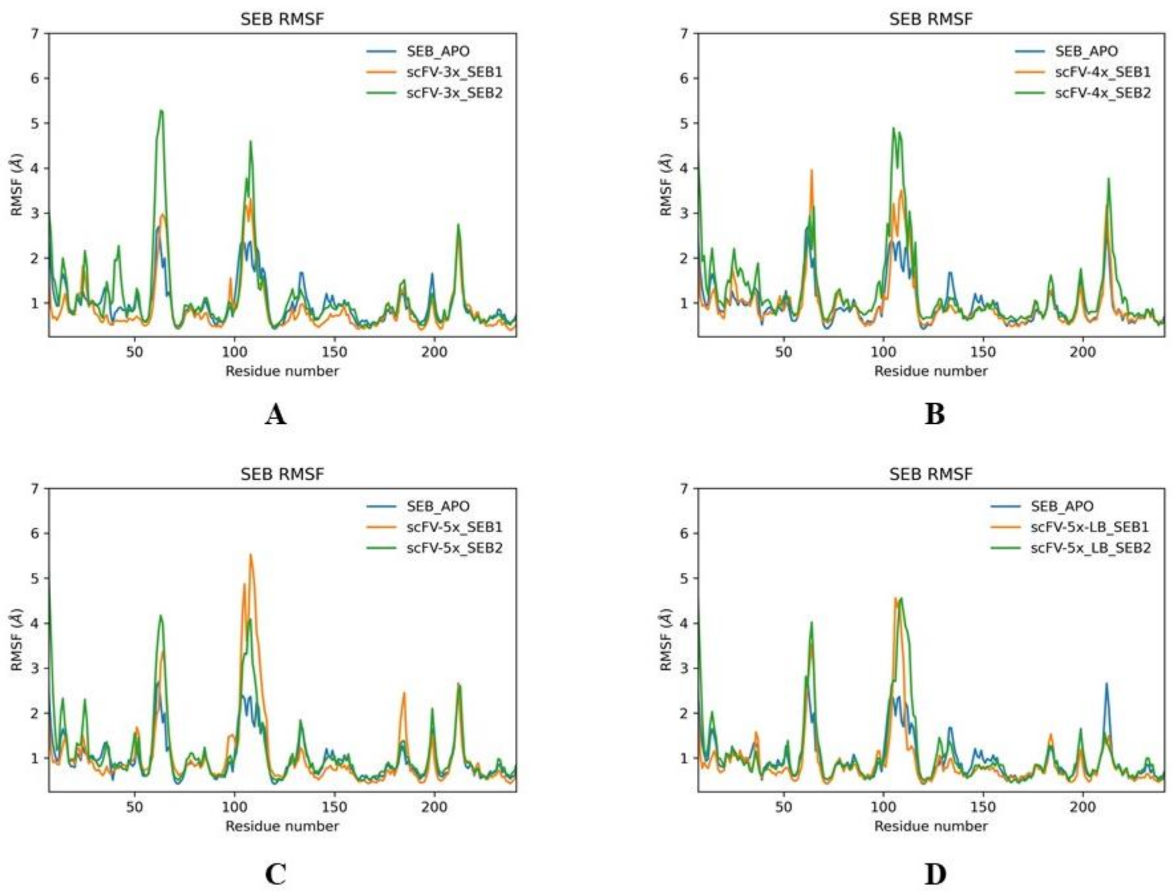


**Supplementary Figure 13. Comparison of the RMSF of SEB from repeated simulations.**

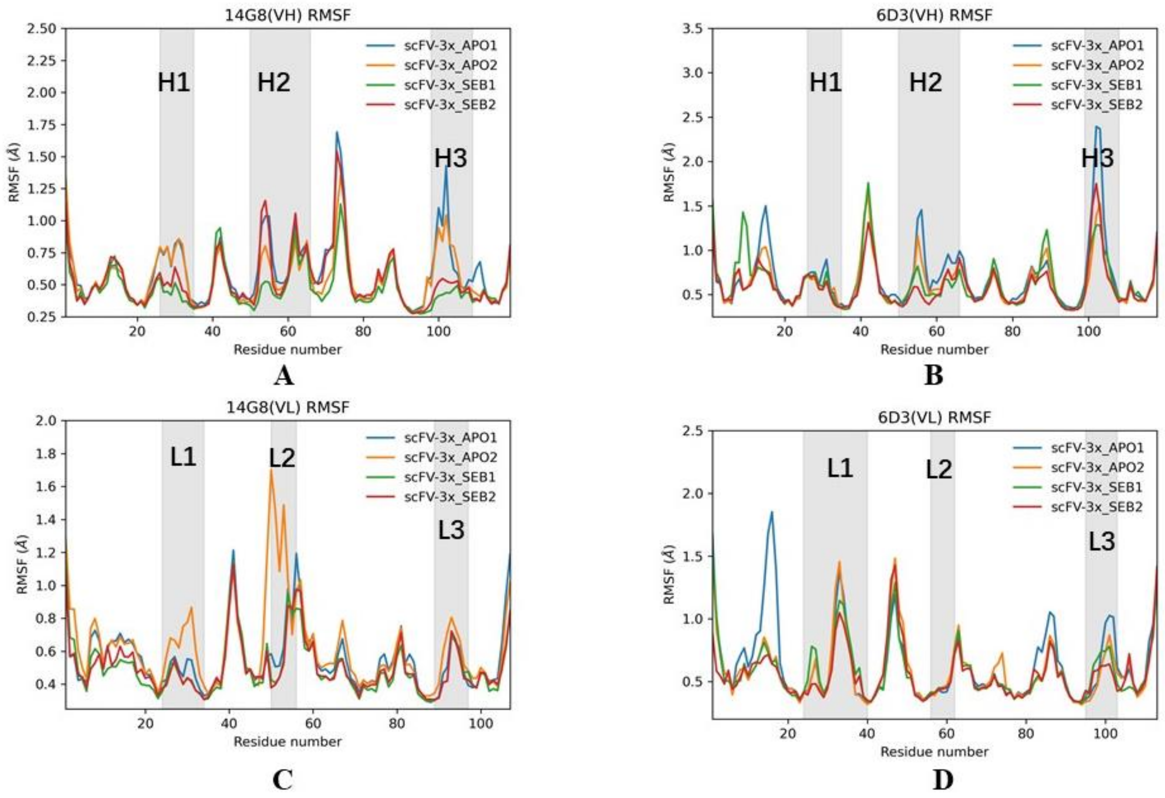


**Supplementary Figure 14. Comparison of the RMSF of scFV from repeated simulations of scFV-3X.**

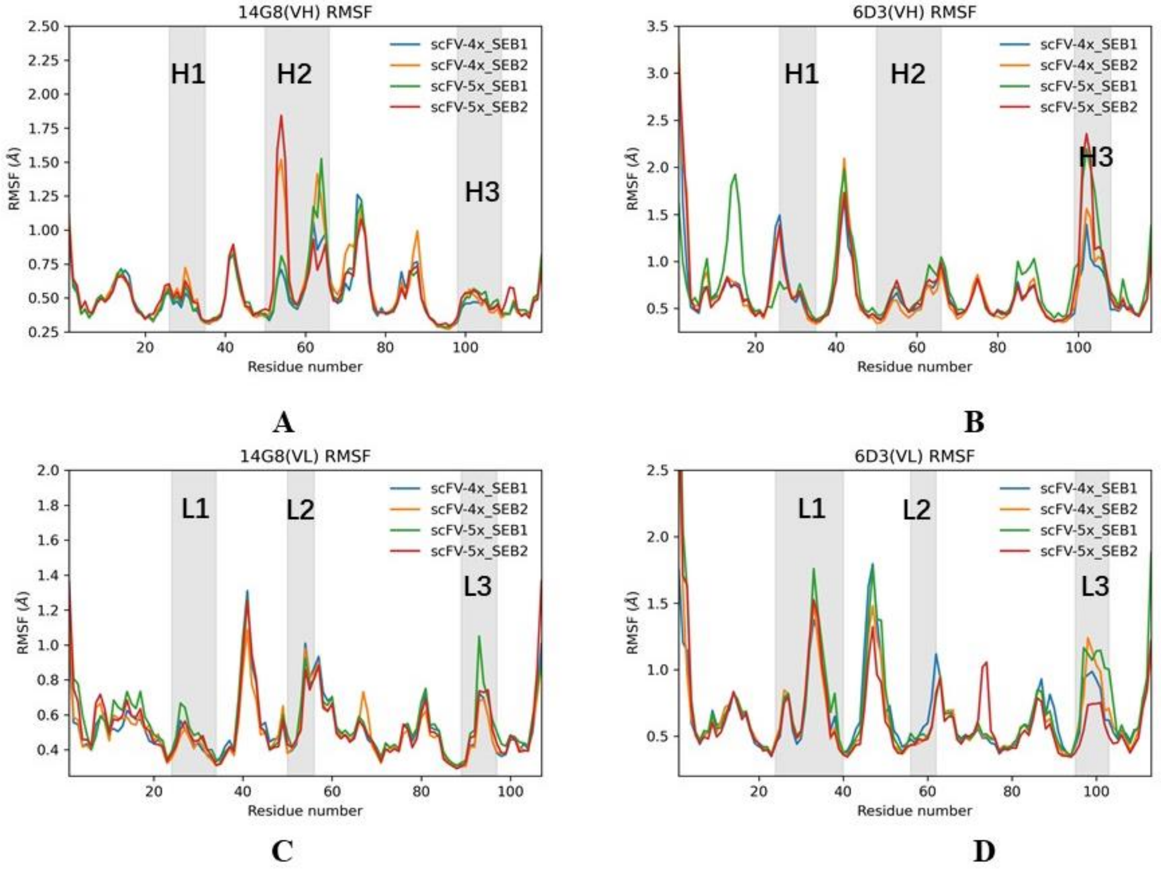


**Supplementary Figure 15. Comparison of the RMSF of scFV from repeated simulations of scFV-4X and scFV-5X.**

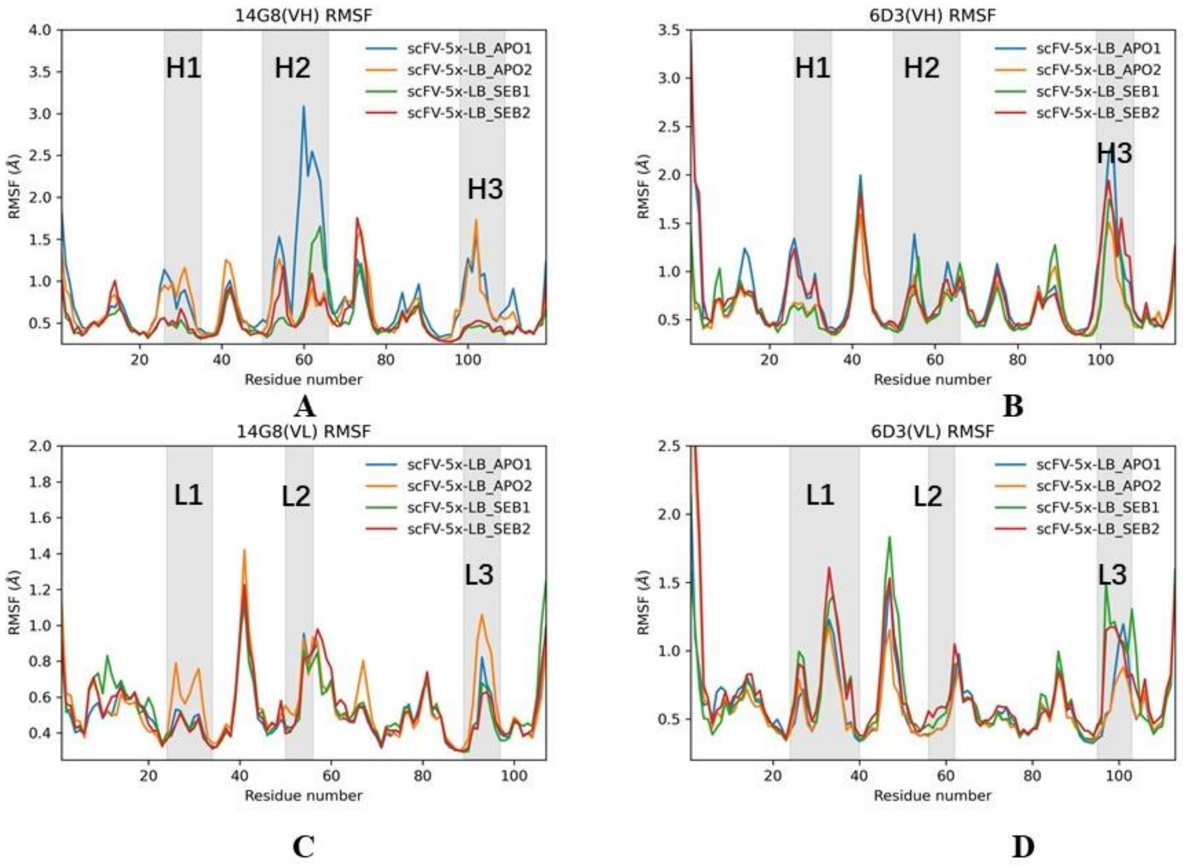


**Supplementary Figure 16. Comparison of the RMSF of scFV from repeated simulations of scFV-5X-LB.**

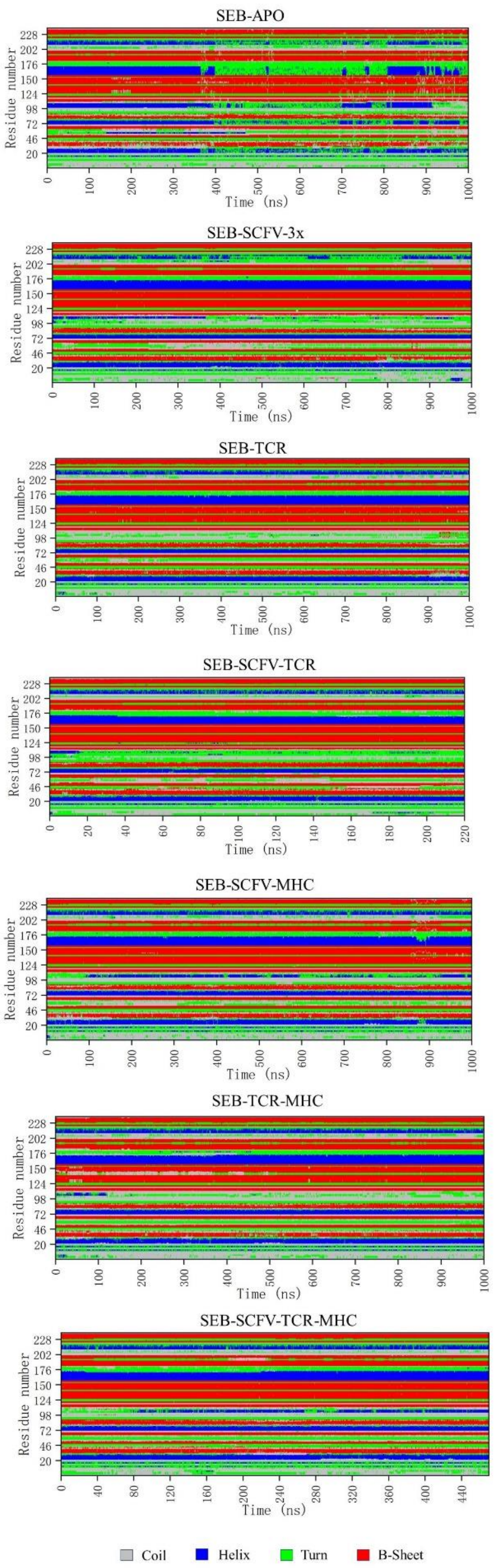


**Supplementary Figure 17. In the SEB apo the loop2 108-110 region was initially modeled as helix and changed to loop during simulation.**
